# Ecological succession in the vaginal microbiota during pregnancy and birth

**DOI:** 10.1038/s41396-020-0686-3

**Published:** 2020-06-02

**Authors:** M. A. Rasmussen, J. Thorsen, M. G. Dominguez-Bello, M. J. Blaser, M. S. Mortensen, A. D. Brejnrod, S. A. Shah, M. H. Hjelmsø, J. Lehtimäki, U. Trivedi, H. Bisgaard, S. J. Sørensen, J. Stokholm

**Affiliations:** 1 COPSAC, Copenhagen Prospective Studies on Asthma in Childhood, Herlev and Gentofte Hospital, University of Copenhagen, Copenhagen, Denmark; 2 Section of Chemometrics and Analytical Technologies, Department of Food Science, University of Copenhagen, Rolighedsvej 26, 1958 Frederiksberg C, Denmark; 3 Novo Nordisk Foundation Center for Basic Metabolic Research, University of Copenhagen, 2100 Copenhagen, Denmark; 4 Department of Biochemistry and Microbiology, Rutgers University, New Brunswick, NJ USA; 5 Departments of Medicine and Microbiology, and the Human Microbiome Program, New York University Langone Medical Center, New York, NY USA; 6 Center for Advanced Biotechnology and Medicine, Rutgers University, New Brunswick, NJ USA; 7 Section of Microbiology, Department of Biology, University of Copenhagen, 2100 Copenhagen, Denmark

**Keywords:** Microbiome, Biodiversity

## Abstract

The mother’s vaginal microbiota represents the first microbes to which a child is exposed when delivered vaginally. However, little is known about the composition and development of the vaginal microbiota during pregnancy and birth. Here, we analyzed the vaginal microbiota of 57 women in pregnancy week 24, 36 and at birth after rupture of membranes but before delivery, and further compared the composition with that of the gut and airways of the 1-week-old child. The vaginal community structure had dramatic changes in bacterial diversity and taxonomic distribution, yet carried an individual-specific signature. The relative abundance of most bacterial taxa increased stepwise from week 24 of pregnancy until birth, with a gradual decline of *Lactobacillus*. Mother-to-child vertical transfer, as suggested by sharing, was modest, with the strongest transfer being for Clostridiales followed by Lactobacillales and Enterobacteriales. In conclusion, late gestation is associated with an increase in maternal vaginal microbiota diversity, and vaginal bacteria at birth only modestly predict the composition of the neonatal microbiota.

## Introduction

A healthy fetus is considered free of microorganisms, and the important initial microbial colonization of the newborn is established by its earliest contact with the environment, especially during and after passage through the birth canal [1, 2]. The human vagina is colonized by a relatively limited set of bacterial taxa, especially during pregnancy, where a shift occurs in the microbiota [3]. This shift leads to a more stable and less diverse composition mainly dominated by a few *Lactobacillus* species, possibly to inhibit the growth of pathogens in this period of increased vulnerability [1, 4].

Anatomically, the female genital tract is arranged as a succession of colonized cavities (cervix and vagina), which communicate with the surroundings, a low pH level is maintained by acid-producing bacteria, comprising mainly of *Lactobacillus* species [5]. The low pH, along with the host immunity, is thought to provide protection against pathogenic colonization of the uterine cavity and fallopian tubes, and thereby protecting the fetus during pregnancy [5, 6].

In women who have given birth, the vaginal microbial populations seem to diversify again to resemble those of nonpregnant women [3]. However, it is not known if this change occurs already during late pregnancy to prepare for birth, or if it is a process starting only after the baby has been born [7].

The objective of the present study was to analyze the natural progression of the maternal vaginal microbial composition during the second half of pregnancy and birth, and its importance for the early neonatal microbial colonization, studying mother–child pairs from the Copenhagen Prospective Studies on Asthma in Childhood_2010_ (COPSAC_2010_) pregnancy and birth cohort [8]. We hypothesized that the microbiota would change during late pregnancy and that the vaginal microbial composition during birth would be among the primary sources of microbes for vertical transfer to the neonatal gut and airways.

## Results

### Shared OTUs between mother’s vagina, and child’s gut and airways

Vaginal birth samples were obtained from 57 women after rupture of membranes but before childbirth. These women also had vaginal samples characterized at pregnancy week 24 (*n* = 56) and week 36 (*n* = 57) and samples were collected from the children (all singletons) of these 57 women 1 week after birth from both feces (*n* = 48) and airways (*n* = 39). The baseline characteristics of the 57 women from whom a vaginal birth sample was obtained were compared with the rest of the cohort comprising 681 pregnant women. These characteristics are detailed in Table S1. The included women were comparable to the remaining cohort in all characteristics apart from a difference in birth season (none of the included women gave birth in the spring) and a trend toward fewer deliveries by cesarean section. All further comparisons were only performed within the set of 57 included women and their children and therefore not affected by these differences.

**Fig. 1:**
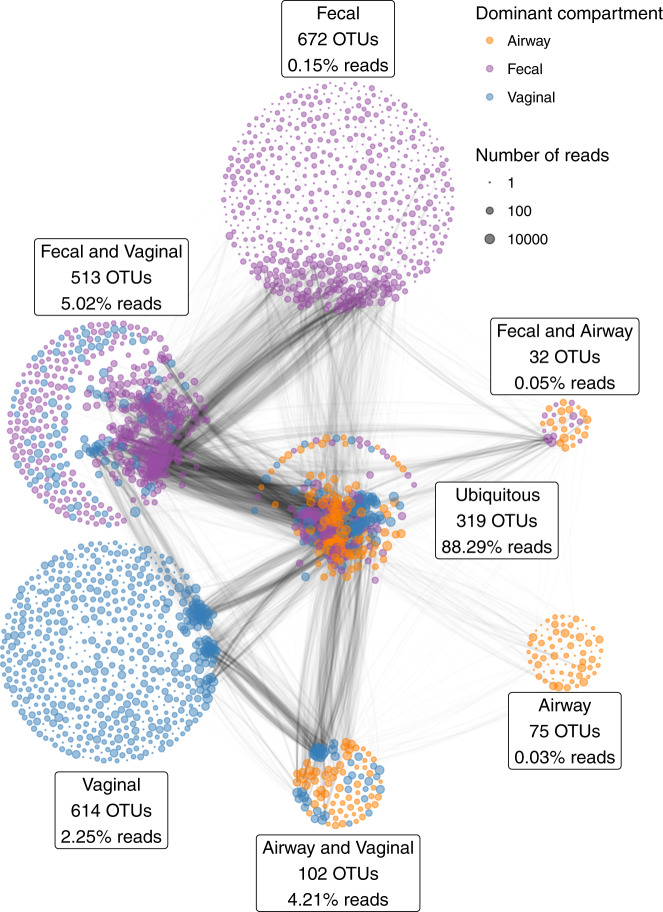
The number of shared and distinct OTUs between vaginal (blue), 1-week fecal (purple) and 1-week airways (yellow) (of a total of 2327 OTUs). The percentages indicate the relative number of reads of the partition; i.e., the 319 ubiquitous OTUs account for a total of 88.3% of the reads. The edges between the OTUs indicate the pairwise correlations (*r*) reflecting the co-occurrence based on a correlation network analysis on each compartment separately, using the highest value among the three compartments. The thickness of the edge corresponds to the maximum pairwise correlation across compartments. Only *r* > 0.2 are plotted.

All samples were characterized by 16S rRNA gene amplicon sequencing of the V4 region. With a median sequencing depth of 43,132 reads in the vaginal samples, 48,764 reads in the fecal samples and 30,949 reads in the airway samples, we identified a total of 2327 unique Operational Taxonomic Units (OTUs); 1548 OTUs in the vaginal samples, 1536 OTUs in the fecal samples, and 528 OTUs in the airway samples. In detail, 839 unique OTUs were identified at pregnancy week 24, 974 OTUs at week 36, and 1071 OTUs at birth. Of all unique OTUs, 319 (13.7%) were ubiquitous OTUs (i.e., present in vagina, gut and airways in at least a single sample) and 647 (27.8%) were shared between two compartments. A total of 88.3% of the reads were from ubiquitous OTUs, while 9.3% were from OTUs shared between any two compartments, leaving only 2.4% as compartment unique reads (Fig. 1). Most of the OTU vs OTU co-occurrence structure occurred in OTUs shared between several compartments, especially ubiquitous OTUs.

### Microbial changes during pregnancy and birth

**Fig. 2:**
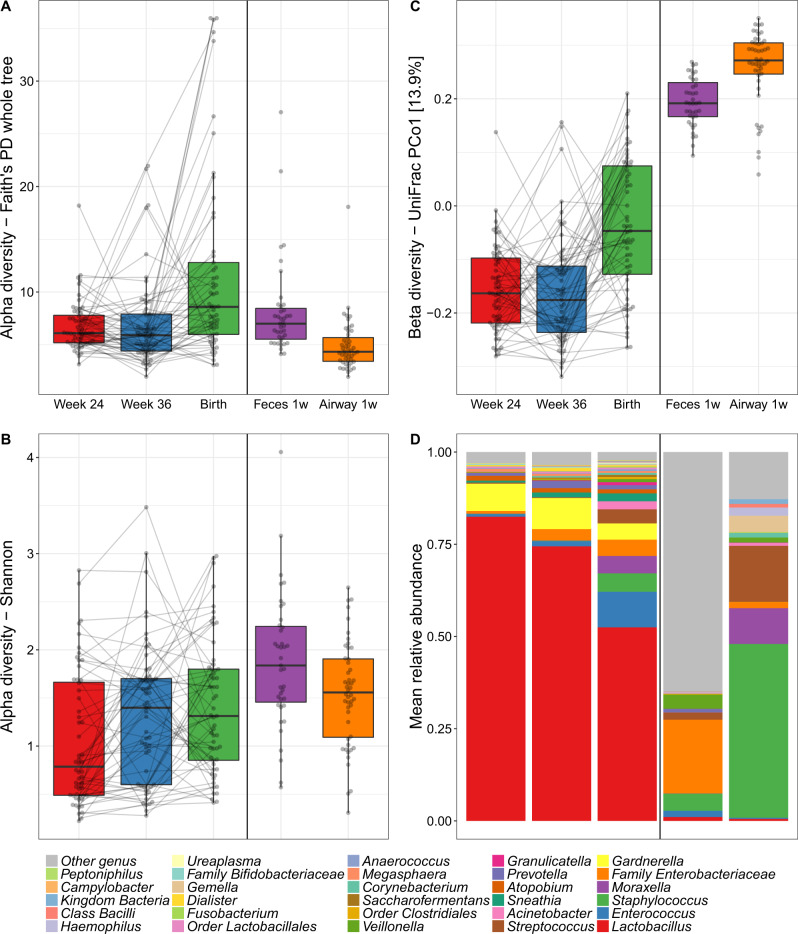
Microbial composition in pregnancy, at birth and after birth. Alpha diversity by **a** Faith’s Phylogenetic Diversity (PD) and **b** Shannon diversity of the vaginal (Week 24, Week 36 and Birth), fecal and airway samples. **c** Primary source of variability between vaginal, fecal and airway samples (PCo1) from ordination by unweighted UniFrac. **d** Relative abundance of the dominant genera.

The median richness per sample was 95 for vaginal, 134 for fecal, and 67 for airway samples. During pregnancy, the median richness per sample was 92 at pregnancy week 24, 85 at week 36, and 117 at birth (see Table S4 for further descriptive details). We investigated variation in vaginal microbial diversity during pregnancy by Faith’s Phylogenetic Diversity (PD) and Shannon diversity indices, representing PD, and a combination of species richness and evenness, respectively. Of the total variation, 35.1% (PD, *p* = 0.10) and 60.5% (Shannon, *p* < 0.001) could be allocated to each individual mother, whereas a general increase in diversity over the three time points during pregnancy contributed with 12.5% (PD, *p* < 0.001) and 4.6% (Shannon, *p* = 0.001) of the variation (Fig. 2a, b). The increase in PD was only observed between pregnancy week 36 (median [IQR], 5.90 [4.41–7.91]) and birth (8.59 [5.99–12.80], *p* < 0.001) with no difference between week 24 (6.10 [5.19–7.79]) and week 36 (*p* = 0.8). Conversely, the increase in Shannon diversity was pronounced between pregnancy week 24 (0.79 [0.49–1.66]) and week 36 (1.40 [0.60–1.70]) (*p* = 0.006) with a non-significant change from week 36 to birth (1.31 [0.85–1.80], *p* = 0.4). We observed a higher Shannon but a lower PD diversity in the children’s 1-week airway and fecal samples compared with the vaginal at birth, which for the fecal samples could reflect an immature composition partly due to ongoing breast-feeding.

We computed unweighted UniFrac ordinations of all three vaginal time-points, as well as the children’s 1-week airway and fecal samples. The main variation in this analysis (Principal Coordinate 1 (PCo1)), was found between the two pregnancy vaginal samples and the children’s fecal and airway samples with the birth samples placed between these two extremes (Fig. 2c). This suggests a development of the perinatal microbiome toward a state taxonomically more resembling the early gut and airways of the child rather than earlier in pregnancy. The same phenomenon was observed when using weighted UniFrac distances which take into account relative abundances (Fig. S1), though this ordination appeared more driven by vaginal-fecal than vaginal-airway differences. The vaginal samples were all dominated by *Lactobacillus*, with declining abundance from pregnancy week 24 until birth (Fig. 2d). The lower relative abundance of *Lactobacillus* resulted in increasing abundance of other genera, including both typical fecal taxa, such as *Enterococcus, Family Enterobacteriaceae*, and *Streptococcus* as well as taxa commonly found in airway and skin such as *Staphylococcus, Moraxella*, and *Streptococcus*. These taxa were all prevalent in the 1-week fecal and airway samples, which had low relative abundances of *Lactobacillus*. These shifts in relative abundances also appeared to be the key drivers behind the observed variation in PCo1 of both unweighted and weighted UniFrac (Figs. S3–S5), while a more divergent set of taxa correlated with subsequent principal coordinates between unweighted and weighted.

Associating the microbiome composition at birth with the birth characteristics (antibiotics during pregnancy, antibiotics during third trimester of pregnancy, antibiotics during birth (mother), season of birth, maternal age at birth, older children in the home, preeclampsia and gestational age at birth) did not reveal any differences regarding alpha- or beta diversity, but suggested a positive correlation between gestational age and genera *Enterococcus and Granulicatella* (*q* value < 0.02) (Table S6).

### Comparison of relative abundances of vaginal microbial taxa

**Fig. 3:**
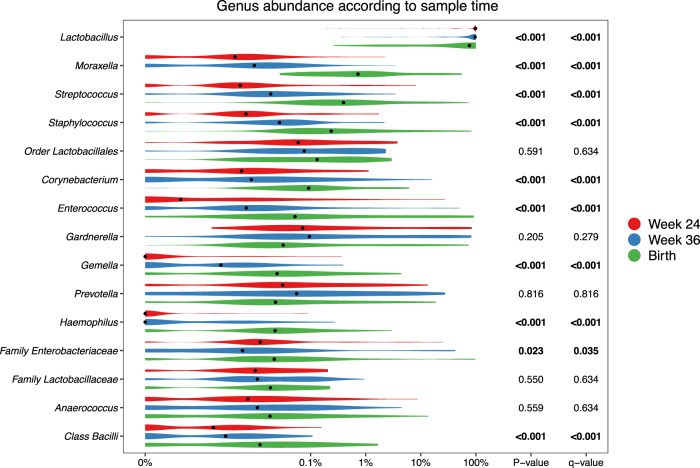
Relative abundances for the top 15 most abundant vaginal taxa at genus level, colored according to time-point. *p*-values correspond to Kruskal–Wallis tests of the relative abundances, with significant values (*p* < 0.05) bolded. A pseudocount (+1e−06) was added to all abundances for the log-scale presentation. False discovery rate *q* values were calculated within these top 15 taxa.

At the genus level, consistent stepwise increases or decreases were seen for the vaginal samples in all the major taxa from pregnancy week 24 through week 36 until birth (Fig. 3 and Table S7 for sensitivity analysis). *Lactobacillus*, by far the most prevalent genus, decreased successively for each time-point. This decrease was not specific to any of the major *Lactobacillus* OTUs but occurred across all of the most commonly identified OTUs, see Figure S6. *Moraxella*, *Streptococcus*, *Staphylococcus*, *Enterococcus*, *Corynebacterium*, and *Gemella* all increased significantly for each successive sample time-point to fill the space corresponding to the decreased *Lactobacillus* abundance. These findings suggest that the perinatal vaginal microbiota does not change suddenly following rupture of membranes or initiation of labor, but rather undergoes a smooth continuous development throughout the last half of pregnancy.

Some taxa (314 specific OTUs) were unique to the birth samples, and were not identified in any of the earlier vaginal samples. Most of these 314 OTUs were only identified in one or few birth samples, but 11 OTUs were found in 5 or more samples. These included four *Moraxella* OTUs, three *Streptococcus*, and some unclassified Bacteria, *Chitinophagaceae*, Bacillales, and Bacilli. However, the combined relative abundance of these unique OTUs in the birth samples was minor (median 0.037% IQR [0.009–0.16%]), see Table S5. The phylogenetic placement of the *Moraxella* and *Streptococcus* OTUs unique to the birth samples are shown in Figs. S7 and S8.

### The individualized vaginal microbiota

**Fig. 4:**
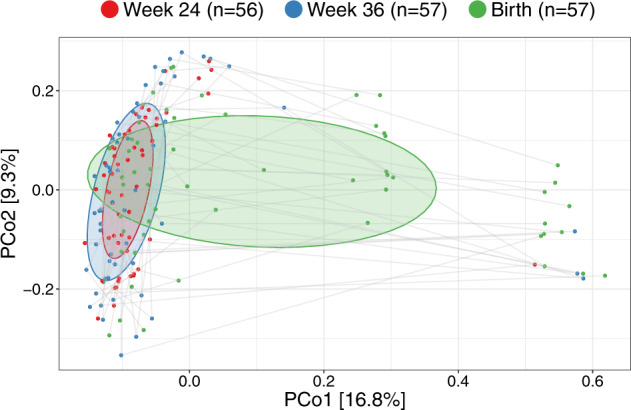
Vaginal succession: Compositional changes in vaginal microbiota. UniFrac ordination of vaginal microbiotas colored according to time-point and joined within individuals. Ellipses demonstrate the mean ± 1SD.

Having established major changes of the vaginal microbiota occurring at birth compared with the two previous time-points, we compared the samples within each woman at the three vaginal time-points. The major direction of variation in the UniFrac PCoA (Fig. 4), as described by Principal Coordinate 1 (PCo1), was the difference between birth (mean PCo1 score 0.13 (SD 0.25)) and both week 36 (−0.05 (0.16), Wilcoxon test *p* < 0.0001) and week 24 (−0.08 (0.09), *p* < 0.0001), while no difference existed between week 36 and week 24 (*p* = 0.63). However, PCo2 was dominated by inter-individual variation, where each mother was a highly significant determinant, as illustrated by the horizontality of lines in Fig. 4. A subanalysis revealed multiple taxa contributing to the variation in PCo1, see Fig. S9. The variation explained in PCo2 by individual mother was 60.3% (linear model, *p* < 0.0001). This large inter-individual as well as time-point variation was confirmed in a joint multivariate model (UniFrac Adonis, time-point *F* value = 7.96, R^2^ = 7.6%, *p* < 0.0001, individual *F* = 1.48, *R*^2^ = 39.5%, *p* < 0.0001). Similar results were found when restricting the analysis to the week 36 and birth samples (UniFrac Adonis, time-point *F* = 6.47, *R*^2^ = 4.8%, *p* < 0.0001, individual *F* = 1.25, *R*^2^ = 53.8%, *p* = 0.0012), week 24 and birth samples (UniFrac Adonis, time-point *F* = 10.98, *R*^2^ = 8.1%, *p* < 0.0001, individual *F* = 1.24, *R*^2^ = 51.3%, *p* = 0.0008) or rarefying to 2000 reads (UniFrac Adonis, time-point *F* = 5.60, *R*^2^ = 5.0%, *p* < 0.0001, individual *F* = 1.95, *R*^2^ = 47.1%, *p* < 0.0001). Collectively, these results reinforce our findings that the vaginal microbiota undergoes major changes at birth, likely including fecal admixture, yet it clearly retains a distinctive individual signature from the earlier pregnancy time-points.

### Vertical transfer from mother to child

We hypothesized that the vaginal microbiota at birth was a major source for the neonatal gut and airway colonization. To investigate this question, each OTU in the vaginal birth samples was tested for transfer to the neonate’s fecal and airway samples at age one week, as well as whether a certain maternal vaginal community structure promoted a certain fecal or airway community structure. The analyses were restricted to the 49 (86%) vaginally delivered children from which 36 and 45 matching neonatal fecal and airway samples were available respectively. From the total of 2327 OTUs, 536 OTUs were found pairwise between vaginal and fecal samples and 346 OTUs between vaginal and airway samples.

**Fig. 5:**
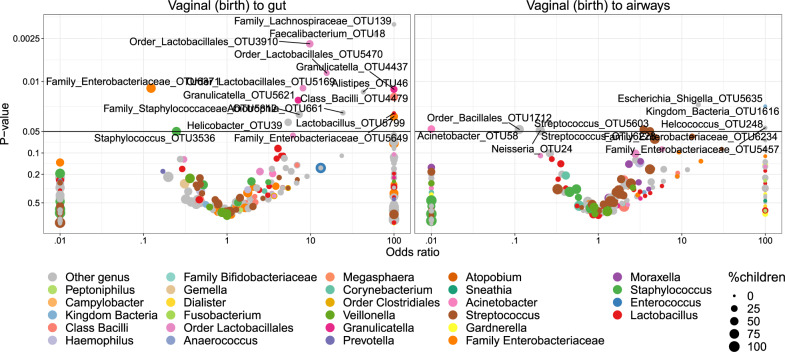
Volcano plot of odds ratios for transfer of individual OTUs from vagina at birth to 1-week feces and airways, respectively. The significant (*p* < 0.05) OTUs above the horizontal line are labeled according to lowest assignable phylogenetic rank. Odds ratios <0.01 or >100 are truncated to these boundaries. Size of the dots indicates the number of children carrying the taxa.

Transfer was first analyzed as presence/absence by Fisher’s exact test. Figure 5 shows volcano plots of the odds of observing a specific OTU in the child given the presence in the vaginal sample at birth. Of these tests, 14 OTUs were significant (*p* < 0.05) for transfer to the gut and 2 OTUs for transfer to the airways. However, none of these significant differences survived correction for multiple testing at false discovery rate (FDR) levels below 0.30. Nevertheless, the distribution of the transfer odds was observed in favor of positive transfer (OR > 1). An enrichment analysis, calculating a weighted ratio (WR) between positive and negative transfer odds revealed ratios between positively and negatively associated OTUs between mother and child samples of 2.7 (*p* = 0.036) and 2.3 (*p* = 0.003) for fecal and airway samples, respectively. This indicates a tendency of transfer that was spread across all OTUs rather than strong transfer of specific OTUs. For robustness analysis of these results see Table S8.

We examined the correlation with the hypothesis that more abundant taxa in the vaginal samples would increase the odds of transfer to the children, but interestingly, higher OTU abundances were negatively correlated with the odds of transfer. However, this was not statistically significant (*p*_gut_ = 0.05, *p*_airways_ = 0.09). Furthermore, the phylogenetic placement of the individual OTUs was examined. Figure S10 shows the odds for vertical transfer on a cladogram, revealing entire branches with only positive associations including OTUs from Lactobacillales (to gut), Enterobacteriales (to both gut and airways) and a clade of Class Bacilli (to gut). These observations were further verified by correlating the phylogenetic placement with the transfer odds, revealing weak although significant relations for transfer to the gut (*R*^2^ = 1.9%, *p* = 0.001) and the airways (*R*^2^ = 1.8%, *p* = 0.03).

Lastly, microbiota similarity was analyzed by means of beta-diversity. Distances between microbiota of mother and child pairs were compared with distances between nonmatching mother and child pairs for both compartments. In concordance with the univariate results, the only diversity measure revealing statistical significance was the number of shared OTUs, as reflected by Jensen–Shannon Divergence (*p*_gut_ = 0.025, *p*_airways_ = 0.006) whereas the other distance metrics were not significant (see Table S9 and Fig. S11 in the Supplementary materials).

## Discussion

Here, we describe changes in microbial populations of the vagina during the second half of pregnancy, ending with dramatic compositional changes at birth, leading to higher diversity and changed taxa representation. The relative abundances of most microbial taxa change as a continuum from week 24 of pregnancy through week 36 and ongoing to term of pregnancy. This suggests a gradual development of the vaginal microbial composition toward birth, where the microbiota diversifies again and has a higher resemblance, in terms of diversity, with that of nonpregnant women [1]. The declining abundance of *Lactobacillus* seems to be a common trait of the vaginal microbial environment late in pregnancy until birth, as similar declining abundances were observed for all the most prevalent *Lactobacillus* OTUs. This natural progression involves an increase in the relative representation of a diversity of bacterial taxa, at the expense of *Lactobacillus*, and results in a conformation more similar to that of the neonatal gut and airway. Despite this observation, we found that the vaginal microbiota at birth only modestly predicts the microbiota in the child at one week of age, suggesting that the neonate receives microbes from other sources than the vagina.

A limitation of the study is that we did not have samples from gut compartment of the mother, which seems to be a larger source for microbial transfer to the child [7]. In addition, vaginal samples before and after pregnancy could have served as helpful reference points for the development during pregnancy. A major strength is the extensive and prospective clinical assessment of environmental exposures in the COPSAC_2010_ population-based mother–child cohort and that all samples were collected using standardized procedures [8]. Furthermore, sequencing was performed in a single laboratory using identical protocols for all samples [9]. Another strength is the possibility to follow the vaginal microbial development in the same women during pregnancy and birth, which allows us to distinguish how much of the changes and variation in the microbial samples was attributed to the individual and how much was attributed to a time-point. We furthermore had samples obtained from children’s feces and airways at one week after birth from the majority of women, which allowed us to analyze transfer, but this analysis is also limited by the resolution inherent to marker-gene derived OTUs. Future studies should employ metagenomic sequencing to resolve bacteria at species- and strain-level to more accurately map the transfer of microbes from mother to child, as has recently been done for the maternal gut microbiome [10–12].

The alpha diversity changed during the last half of pregnancy. The Shannon diversity index increased significantly between week 24 and 36 driven by an increase in evenness, with only a slight non-significant additional increase to birth. In contrast, Faith’s PD showed only a significant increase between week 36 and birth, as an effect of the colonization of new phylogenetically distinct bacteria in the birth samples (Fig. 4). The overall composition, measured as UniFrac distances, did not change much from pregnancy week 24 to 36 which indicates an overall stability with respect to the set of taxa present in this part of pregnancy; however, a large shift appeared at birth. The results from both diversity and taxonomic analyses suggest that the vaginal microbiota did not change all at once following rupture of membranes but instead seems to undergo a continuous development throughout the last part of pregnancy. Most taxa besides *Lactobacillus* were found in higher abundances in the birth samples and phylogenetically different microbes more commonly associated with other microbial compartments, such as gut, airways and skin were introduced here. Pregnancy is a unique immune condition modulated during three distinct immunological phases: the first trimester of pregnancy is mainly a pro-inflammatory phase [13], followed by induction of an anti-inflammatory state until the final phase when there is renewed inflammation [14] culminating in contraction of the uterus, expulsion of the baby and rejection of the placenta [15]. Consistently, differences in cytokines reflect the sensitivity to infectious diseases, higher during the first half of the pregnancy and this risk gradually declines during the second half, as observed for malaria infection [16]. These changes in immune status as well as hormonal fluctuations[17] during this period correlate with changes in the vaginal microbiome, and they could be causally related. It can be speculated that nature prepares the woman to give birth and transfer a more diverse composition to the child. Here, the vaginal microbiota is believed to be among the primary sources for microbial colonizers, which happens during the passage through the birth canal [1, 2]. We did not observe any clinical parameters strongly associated with the microbiome composition at birth, however higher gestational age was associated with a higher abundance of *Enterococcus*, which supports the differences observed between week 36 and birth.

We observed signs of vertical transfer of microbes from mother to child, with slightly stronger results to the gut of the children compared with the airways, which is in accordance with a previous study on gut and airway composition after cesarean section, a setting where the vertical transfer is interrupted [18]. The study does not support that the entire community structure of the maternal vaginal sample was transferred, nor that specific transfer prone taxa existed, but rather that taxa present in the maternal vaginal sample at birth were more often found in her own child compared with other children. High taxa abundance in the vagina of the mothers did not lead to higher rates of vertical transfer for these which could resemble that compartment specific microbes are less shared between dyads compared with the ubiquitous microbes. Here, consistent associations were found for order Clostridiales (transfer to gut) and to some extent Enterobacteriales (to both gut and airways) and Lactobacillales (transfer to gut). However, these results only indicate a modest influence of the vaginal microbiota on the composition of the microbiota in the newborn child. This lesser role of microbial transfer from vagina to gut has previously been described [7].

Our results showed a specific ecological succession in the vaginal microbiota during pregnancy and birth, leading to a decrease in *Lactobacillus* and increase in PD. However, as we found limited evidence for transfer of the birth microbiota to the offspring, it seems unlikely that this phenomenon has evolved to ensure a healthy neonatal colonization. Further studies are needed to elucidate if the development in vaginal birth microbiota serves another purpose or if the phenomenon is simply a byproduct of physiological changes related to pregnancy and birth.

In conclusion, late gestation was associated with an increase in maternal vaginal microbiota diversity as a continuum from pregnancy week 24 over week 36 to birth, and vaginal bacteria at birth only modestly predict the composition of the neonatal microbiota.

## Methods

### Study population

The COPSAC_2010_ cohort is an ongoing Danish cohort study of 738 unselected pregnant women and their 700 children followed from pregnancy week 24 in a protocol described in detail [8]; the study began enrollment in 2008 which was completed by 2011. Data validation and quality control follow the guidelines for good clinical practice [8]. Data were collected during the visits to the clinical research unit, stored in a dedicated online database, double-checked against source data, and subsequently locked.

### Ethics

The study was conducted in accordance with the guiding principles of the Declaration of Helsinki and was approved by the Local Ethics Committee (H-B-2008-093), and the Danish Data Protection Agency (2015-41-3696). Both parents gave written informed consent before enrollment [8].

### Samples and preparation

Vaginal samples from asymptomatic pregnant women were collected at the week 24 and 36 of pregnancy in the COPSAC clinic and at birth after rupture of membranes but before passage of the child either by the woman herself or by the midwife; detailed written instructions were provided. The women were sampled from the posterior vaginal fornix using flocked swabs (ESWAB flocked regular, SSI Diagnostica, Hillerød, Denmark). Hypopharyngeal airway aspirates were obtained at age one week from the child using a soft suction catheter passed through the nose into the hypopharynx in the COPSAC clinic as described in detail [19]. Fecal samples were also collected from the child at age 1 week, either at the research clinic or by the parents at home, using detailed written instructions. All samples were frozen at −80 °C within 24 h. Each fecal sample was mixed on arrival with 10% vol/vol glycerol broth (Statens Serum Institut, Copenhagen, Denmark). DNA was extracted using MoBio PowerSoil kits on an epMotion 5075, amplified using a two-step PCR reaction with 16S rRNA gene primers 515F and 806R that flank the V4 region, and sequenced using the v2 kit (PE250bp reads) on the MiSeq platform (Illumina Inc., San Diego CA), as described [9, 20]. For details on the extraction, sequencing and variance contribution from each source see Tables S2, S3 and Fig. S2, which suggests larger biological variation compared with wetlab procedure. Due to confounding between extraction kit, sequencing run and pregnancy time-point, sensitivity analysis were conducted comparing timepoint differences within a single sequencing run and extraction kit respectively (see Table S7).

### Covariates

Information was obtained during the scheduled visits to the COPSAC clinic, and included maternal age at birth, smoking and alcohol use during pregnancy, education, household income, antibiotics received during pregnancy, preeclampsia, gestational diabetes, delivery type, gestational age, asthma in the mother, hospitalization of the child after birth, antibiotics prescribed to the child, household pets, and number of older children at home. Information on intrapartum antibiotics and antibiotic use during pregnancy and childhood was validated against national registries [21].

### Data processing

Fastq-files demultiplexed by the MiSeq Controller Software were trimmed for amplification primers, diversity spacers, and sequencing adapters, mate-paired and quality filtered (USEARCH v7.0.1090) [22]. UPARSE [23, 24] was used for Operational Taxonomic Unit (OTU) clustering at 97% identity as recommended, in particular removing singletons after dereplication. Chimera checking was performed with UCHIME [25] against Mothur’s supplied version of the RDP9 PDS database. Representative sequences were classified at 0.8 confidence threshold (Mothur v1.25.0 wang function) [26]. FastTree [27] and Mothurs align.seq function [28] were used to construct a phylogenetic tree. Alignments were built with reference to the 2013 version of Greengenes [29]. The laboratory workflow and bioinformatics pipeline has been described [9, 20].

### Statistics

All data analysis was performed in the statistical software package R version 3.3.0, with the package phyloseq [30] to handle microbiome data. Samples below 2000 reads were omitted from the analysis. Chi-square test, Student’s *t* test or Wilcoxon rank-sum test was used for analyzing simple associations in the baseline characteristics of the cohort with reference to differences between included and excluded participants and prenatal, perinatal, social, and lifestyle-related variables. Correlations between OTUs were calculated using the sparse correlations for compositional data (sparCC) correlation metric [31] within each compartment and combined as the maximum across the three compartments. Values below 0.2 were pruned from the set, before plotting a Fruchterman–Reingold network in each sub-category (each compartment or combinations thereof).

The α-diversity measures of richness (number of unique OTUs), Faith’s Phylogenetic Diversity [32] and Shannon diversity index were used as measures of the intra-individual diversity, and the inter-individual (β-) diversity was computed as unweighted UniFrac [33] distances. α-diversity (PD and Shannon) were compared by an ANOVA model splitting the variation into pregnancy week and individual mother. The relative abundances at genus level were compared between time-points using the paired Wilcoxon rank-sum test (dichotomous) or Kruskal–Wallis Test (>2 levels) and differences in β-diversity were visualized with principal coordinates analysis (PCoA) plots and tested for inference using permutational multivariate analysis of variance (PERMANOVA) (Adonis from the package vegan, with 999 permutations) [34, 35] or with linear regression for each principal coordinate against time-points and influence of the specific mother.

Vertical transfer of the vaginal microbiota at birth to the infant gut and airways in the 1-week-old child was restricted to vaginal birth only, and analyzed by univariate Fisher’s exact test for presence/absence of taxa in both compartments (only taxa with a presence/absence range defined as between 1 and n-1 non-zero counts in the vaginal birth samples, as well as the 1-week compartments, are susceptible for this analysis. n is the number of children/mother pairs). An enrichment analysis were conducted post-hoc using the individual OTU analysis results as input. That is: For each compartment, the univariate OTU-wide comparisons were analyzed for enrichment of positive associations by calculation of a WR between positive (OR > 1) and negative (OR < 1) associations, defined as:

$$WR = \frac{\sum_{i \in OR > 1}{log(OR_i) \cdot log(p_i)}}{\sum_{i \in OR < 1}{-log(OR_i) \cdot log(p_i)}}$$

Under the null hypothesis of no association the expectation of WR is 1. To test this hypothesis, random permutation is performed scrambling the connection between mother and child, followed by individual OTU wise Fisher exact test and calculation of WR. This is done 999 times to construct a null distribution in which the observed WR is tested. In addition, the maternal abundance for each OTU was investigated as a descriptor for the odds of transfer. Transfer was analyzed as distances between mother–child pairs for a range of different beta diversity measures, comparing these with randomly matched mothers and children by permutation testing (999 permutations). A significance level of 0.05 was used in all analyses. Visualizations were done using ggplot2 (ver 3.1.0) and cladogram was drawn based on phylogenetic distance using the R package ggtree [36].

### Data and code

The sequences are available at short read archive under project number PRJNA595451 (maternal vagina during birth, and fecal and airways of the 1-week-old offspring), PRJNA576765 (maternal vaginal data from pregnancy week 24) and RJNA579012 (maternal vaginal data from pregnancy week 36). Further, a github (https://github.com/mortenarendt/MBtransfeR) encompasses an R-packages as well as documents the analysis, including the phyloseq object including anonymized data.

## Supplementary information

Supplemetal Material
