# Supplementary material for: Ecological succession in the vaginal microbiota during pregnancy and birth
Source: ISME J. 2020 Jun 2;14(9):2325–35. doi: 10.1038/s41396-020-0686-3 (PMC7609337; doi:10.1038/s41396-020-0686-3)
Supplement: Supplementary file 1 — Supplemetal Material [file 41396_2020_686_MOESM1_ESM.pdf]

Ecological succession in the vaginal microbiota during pregnancy and birth - *Supplementary material*

Rasmussen, M.A.[1+2 $], Thorsen, J.[1+3 $], Dominguez-Bello, M.G.[4], Blaser, M. J.[5+6], Mortensen, M.S.[7], Brejnrod, A.D.[3+7] Shah, S.A.[1], Hjelmsoe, M.H.[1], Lehtimäki, J.[1], Trivedi, U.[7], Bisgaard H.[1], Sørensen S.J.[7#], Stokholm J.[1#]

<u>Affiliation:</u>
1) COPSAC, Copenhagen Prospective Studies on Asthma in Childhood, Herlev and Gentofte Hospital, University of Copenhagen, Copenhagen, Denmark
2) Section of Chemometrics and Analytical Technologies, Department of Food Science, University of Copenhagen, Rolighedsvej 26, 1958 Frederiksberg C, Denmark
3) Novo Nordisk Foundation Center for Basic Metabolic Research, University of Copenhagen, 2100 Copenhagen, Denmark
4) Department of Biochemistry and Microbiology, Rutgers University, New Brunswick, NJ, USA
5) Departments of Medicine and Microbiology, and the Human Microbiome Program, New York University Langone Medical Center, New York, USA.
6) Center for Advanced Biotechnology and Medicine, Rutgers University, New Brunswick, NJ, USA
7) Section of Microbiology, Department of Biology, University of Copenhagen, 2100 Copenhagen, Denmark

[$]Contributed equally, [#]Contributed equally

Table S1 *- Baseline characteristics for women participating in the study. Numbers in table refer to either N (%) or mean (SD), depending on the variable. P-values correspond to Chi-square test (categorical variables) or t-tests (continuous variables).*

	**Included**	**Remaining**	**p-value**
	57	681	
Cesarean section, N (%)	6 (10.9)	144 (22.5)	0.067
Hospitalized at birth, N (%)	4 (7.3)	74 (11.6)	0.598
Non-caucasian, N (%)	2 (3.6)	27 (4.2)	1.000
Smoking in pregnancy, N (%)	7 (12.7)	47 (7.3)	0.242
Preeclampsia, N (%)	3 (5.5)	27 (4.2)	0.933
Gestational diabetes, N (%)	0 (0.0)	16 (2.5)	0.471
Intrapartum antibiotics, N (%)	13 (23.6)	209 (32.8)	0.212
Nulliparity, N (%)	22 (40.0)	297 (46.4)	0.439
Male child, N (%)	30 (54.5)	326 (50.9)	0.709
Birth season, N (%)			<0.001
Winter	12 (21.8)	201 (31.4)	
Spring	0 (0.0)	185 (28.9)	
Summer	7 (12.7)	141 (22.0)	
Fall	36 (65.5)	113 (17.7)	
Maternal age at birth, mean (SD), years	32.46 (4.24)	32.26 (4.37)	0.750
Gestational age, mean (SD), weeks	40.20 (1.10)	39.85 (1.67)	0.127
Antibiotics in pregnancy, N (%)	25 (45.5)	228 (35.7)	0.194
Household annual income, N (%)			0.137
Low (below 50.000 Euro)	1 (1.8)	66 (10.3)	
Medium (50.000 – 110.000 Euro)	28 (50.9)	340 (53.1)	
High (above 110.000 Euro)	26 (47.3)	233 (36.4)	
Maternal educational level, N (%)			0.979
Low (elementary school or college graduate)	4 (7.3)	51 (8.0)	
Medium (tradesman or medium length)	36 (65.5)	412 (64.4)	
High (master's degree)	15 (27.3)	177 (27.7)	

Table S2 - Wetlab protocol (N) for the five sample types on lot number of extraction kit and extraction tray.

**Lot number Extraction Kit**	**PH13 C27**	**PM13A 29**	**PM13A 30**	**PM13 C27**					**PM13 H15**			
**Extraction tray**	**33**	**19**	**18**	**49**	**50**	**51**	**34**	**32**	**56**	**57**	**58**	**59**
**Fecal w1**	29						1	9				
**Trach w1**		24	24									
**Vaginal w24**				12	39	5						
**Vaginal w36**									2	44	11	
**Vaginal birth**												57

Table S3 - *Sequencing run and the five sample types (N).*

**Sequencing Run**	**Sr1**	**Sr2**	**Sr3**	**Sr4**	**Sr5**	**Sr6**	**Sr7**	**Sr8**	**Sr9**	**Sr10**	**Sr11**
**Fecal w1**		9				23	7				
**Trach w1**	24				8					16	
**Vaginal w24**			39		7				3		7
**Vaginal w36**				44	2			11			
**Vaginal birth**				57							

Table S4 *- Summary statistics (median (25% quartile; 75% quartile)) for compartment and sampling time point. N = number of samples, library size = number of reads, #OTUs = number of observed OTUs, PD = Faith's Phylogenetic Diversity, Shannon = Shannon diversity.*

	**N**	**library size**	**#OTUs**	**PD**	**Shannon**
**Fecal w1**	39	48764 (17513;82239)	134 (116;210)	7.00 (5.53;8.45)	1.84 (1.46;2.24)
**Trach w1**	48	30948 (19896;53692)	67 (52;83)	4.34 (3.43;5.68)	1.56 (1.09;1.91)
**Vaginal w24**	56	52900 (38058;65348)	92 (76;122)	6.10 (5.19;7.79)	0.79 (0.49;1.66)
**Vaginal w36**	57	41270 (27535;59467)	85 (62;118)	5.90 (4.41;7.91)	1.40 (0.60;1.70)
**Vaginal birth**	57	39708 (32062;49646)	117 (87;137)	8.59 (5.99;12.8)	1.31 (0.85;1.80)

Table S5 *- Unique OTUs found in vaginal birth samples, but not in pregnancy week 24 or 36 samples (OTUs identified in ≥5 samples).*

**Taxa name**	**Positive (N)**	**Mean relative abundance**	**Reads (N)**
Streptococcus_OTU6228	14	0.0021	81
Moraxella_OTU4678	13	0.0007	28
Kingdom_Bacteria_OTU553	12	0.0069	245
Moraxella_OTU3511	11	0.0004	14
Streptococcus_OTU6224	10	0.0009	33
Moraxella_OTU4650	8	0.0004	16
Streptococcus_OTU5596	7	0.0046	186
Order_Bacillales_OTU3316	7	0.0027	121
Family_Chitinophagaceae_OTU990	7	0.0014	64
Class_Bacilli_OTU4895	5	0.0009	38
Moraxella_OTU6681	5	0.0003	9

Table S6 *- Association between major clinical variables and the vaginal microbiota at birth, quantified as* ***A)*** *Alpha diversity (Shannon index, linear models),* ***B)*** *Beta diversity (unweighted and weighted UniFrac) and* ***C)*** *Differential abundance tests of the top 15 most abundant taxa vs. gestational age (spearman correlation test) and any antibiotics administered during birth or in the third trimester (Kruskal-Wallis test). The only significant findings were associations between gestational age at birth and the two closely related taxa Enterococcus and Granulicatella (Between-taxa relative abundance spearman correlation 0.78, p = 1.24e-12). q values are calculated using False Discovery Rate (fdr) control.*

S6A

Clinical information	estimate	std.error	conf.low	conf.high	p.value	q.value
Antibiotics during pregnancy	0.11	0.19	-0.27	0.49	0.57	0.85
Antibiotics during third trimester of pregnancy	0.05	0.23	-0.41	0.50	0.84	0.86
Antibiotics during birth (mother)	-0.04	0.22	-0.48	0.40	0.86	0.86
Season of birth	0.01	0.08	-0.14	0.17	0.86	0.86
Maternal age at birth	0.06	0.02	0.02	0.10	0.01	0.06
Older children in the home	-0.14	0.19	-0.52	0.25	0.48	0.85
Preeclampsia	-0.90	0.40	-1.69	-0.10	0.03	0.13
Gestational age at birth	0.06	0.09	-0.12	0.23	0.52	0.85

S6B

Clinical information	Metric	F stat	R^2	p.value
Preeclampsia	UniFrac	0.66	0.012	0.89
Maternal age at birth	UniFrac	0.52	0.010	0.96
Older children in the home	UniFrac	1.51	0.028	0.11
Season of birth	UniFrac	1.35	0.025	0.15
Antibiotics during birth (mother)	UniFrac	0.77	0.014	0.68
Antibiotics during pregnancy	UniFrac	0.75	0.014	0.69
Antibiotics during third trimester of pregnancy	UniFrac	1.33	0.025	0.17
Gestational age at birth	UniFrac	1.06	0.020	0.33
Preeclampsia	wUniFrac	0.65	0.012	0.67
Maternal age at birth	wUniFrac	1.21	0.022	0.26
Older children in the home	wUniFrac	1.13	0.021	0.28
Season of birth	wUniFrac	0.34	0.006	0.94
Antibiotics during birth (mother)	wUniFrac	0.55	0.010	0.75
Antibiotics during pregnancy	wUniFrac	0.51	0.009	0.79
Antibiotics during third trimester of pregnancy	wUniFrac	0.38	0.007	0.91
Gestational age at birth	wUniFrac	0.57	0.011	0.70

S6C

**Clinical information**	**Genus**	**Method***	**estimate**	**p.value**	**q.value**
Antibiotics during birth (mother)	Acinetobacter	KW		0.94	0.94
Antibiotics during birth (mother)	Atopobium	KW		0.44	0.93
Antibiotics during birth (mother)	Enterococcus	KW		0.74	0.93
Antibiotics during birth (mother)	Family_Enterobacteriaceae	KW		0.94	0.94
Antibiotics during birth (mother)	Gardnerella	KW		0.57	0.93
Antibiotics during birth (mother)	Granulicatella	KW		0.33	0.93
Antibiotics during birth (mother)	Lactobacillus	KW		0.51	0.93
Antibiotics during birth (mother)	Moraxella	KW		0.86	0.94
Antibiotics during birth (mother)	Order_Clostridiales	KW		0.13	0.93
Antibiotics during birth (mother)	Prevotella	KW		0.19	0.93
Antibiotics during birth (mother)	Saccharofermentans	KW		0.57	0.93
Antibiotics during birth (mother)	Sneathia	KW		0.17	0.93
Antibiotics during birth (mother)	Staphylococcus	KW		0.20	0.93
Antibiotics during birth (mother)	Streptococcus	KW		0.74	0.93
Antibiotics during birth (mother)	Veillonella	KW		0.67	0.93
Antibiotics during third trimester of pregnancy	Acinetobacter	KW		0.50	0.93
Antibiotics during third trimester of pregnancy	Atopobium	KW		0.38	0.93
Antibiotics during third trimester of pregnancy	Enterococcus	KW		0.48	0.93
Antibiotics during third trimester of pregnancy	Family_Enterobacteriaceae	KW		0.55	0.93
Antibiotics during third trimester of pregnancy	Gardnerella	KW		0.78	0.93
Antibiotics during third trimester of pregnancy	Granulicatella	KW		0.81	0.94
Antibiotics during third trimester of pregnancy	Lactobacillus	KW		0.70	0.93
Antibiotics during third trimester of pregnancy	Moraxella	KW		0.61	0.93
Antibiotics during third trimester of pregnancy	Order_Clostridiales	KW		0.85	0.94
Antibiotics during third trimester of pregnancy	Prevotella	KW		0.51	0.93
Antibiotics during third trimester of pregnancy	Saccharofermentans	KW		0.76	0.93
Antibiotics during third trimester of pregnancy	Sneathia	KW		0.60	0.93
Antibiotics during third trimester of pregnancy	Staphylococcus	KW		0.40	0.93
Antibiotics during third trimester of pregnancy	Streptococcus	KW		0.89	0.94
Antibiotics during third trimester of pregnancy	Veillonella	KW		0.01	0.22
Gestational age at birth	Acinetobacter	Spearman	0.07	0.63	0.93
Gestational age at birth	Atopobium	Spearman	-0.08	0.56	0.93
**Gestational age at birth**	**Enterococcus**	**Spearman**	**0.48**	**0.0002**	**0.01**
Gestational age at birth	Family_Enterobacteriaceae	Spearman	0.09	0.52	0.93
Gestational age at birth	Gardnerella	Spearman	0.14	0.30	0.93
**Gestational age at birth**	**Granulicatella**	**Spearman**	**0.45**	**0.0005**	**0.01**
Gestational age at birth	Lactobacillus	Spearman	-0.24	0.07	0.83
Gestational age at birth	Moraxella	Spearman	-0.08	0.56	0.93
Gestational age at birth	Order_Clostridiales	Spearman	-0.14	0.32	0.93
Gestational age at birth	Prevotella	Spearman	-0.14	0.30	0.93
Gestational age at birth	Saccharofermentans	Spearman	-0.06	0.65	0.93
Gestational age at birth	Sneathia	Spearman	0.07	0.60	0.93
Gestational age at birth	Staphylococcus	Spearman	0.05	0.72	0.93
Gestational age at birth	Streptococcus	Spearman	-0.04	0.78	0.93
Gestational age at birth	Veillonella	Spearman	0.02	0.90	0.94

* KW = Kruskal-Wallis rank sum test, Spearman = Spearman's rank correlation rho

***Table S7** – Sensitivity analysis of vaginal development during pregnancy to birth with respect to sequencing run as well as extraction kit. Top 10 most abundant and top 10 most present taxa On genus level, as well as the diversity measures PCo1, PD and Shannon. "All" refers to all women with data from three timepoints, whereas "Single seq run" refers to paired samples from pregnancy week 36 and birth from 44 women from sequencing run 4 (**Sr4**), and "Single extraction kit lot" refers to all samples but only from pregnany week 36 and birth from from extraction kit lot **PM13H15**. n indicates the number of samples for this analysis. Reported is p values based on Kruskal Wallis test for difference between timepoints. **Mean RA** refers to the mean relative abundance within the vaginal samples.*

Genus	Mean RA	All (n = 3 x 56)	PM13H15 (n = 2 x 57)	SR_4 (n = 2 x 44)
**Atopobiu**	0.012	0.692	0.436	0.966
**Dialister**	0.004	0.289	0.105	0.200
**Enterococcus**	0.039	<0.001	<0.001	0.002
**Family_Enterobacteriaceae**	0.027	0.037	0.011	0.026
**Family_Lactobacillaceae**	0.0005	0.544	0.871	0.590
**Gardnerella**	0.067	0.160	0.131	0.246
**Lactobacillus**	0.70	<0.001	<0.001	0.004
**Moraxella**	0.016	<0.001	<0.001	<0.001
**Order_Lactobacillales**	0.0046	0.497	0.803	0.835
**Prevotella**	0.012	0.908	0.648	0.652
**Sneathia**	0.013	0.086	0.836	0.343
**Staphylococcus**	0.017	<0.001	<0.001	<0.001
**Streptococcus**	0.014	<0.001	<0.001	<0.001
**PCo1 (unifrac)**		<0.001	<0.001	<0.001
**PD**		<0.001	<0.001	0.001
**Shannon**		0.011	0.406	0.350

***Table S8** - Weighted transfer Ratios (WR) from vaginal birth microbiome to fecal- and airway microbiome age one week, based on all data as well as rarefied to lowest common depth (2325 reads per sample). notu = number of test-able otus for each analysis, pv = permutation p-value for WR statistics (999 permutations).*

Compartment	rarefy	notu	WR	pv
**Fecal**	No	536	2.69	0.036
**Fecal**	Yes	222	6.03	0.004
**Airway**	No	346	2.25	0.003
**Airway**	Yes	156	3.72	0.001

Table S9 *– Beta-diversity comparison between vaginal microbiotas at birth and 1-week fecal and 1-week airway in children born by vaginal delivery. P values and distances of matching and non-matching mother-child pairs are computed by random permutation (n = 999)*

		**Fecal (dyads, n = 36)**					**Airway (dyads, n = 45)**				
	**Metric**	**Distance**					**Distance**				
		**Dyad**		**Random**			**Dyad**		**Random**		
		**mean**	**SD**	**mean**	**SD**	**p-value**	**mean**	**SD**	**mean**	**SD**	**p-value**
**Absence/ presence**	**Jaccard**	0.982	0.039	0.990	0.025	0.076	0.955	0.094	0.966	0.077	0.110
	**Binomial**	176	68	179	71	0.028	107	38	109	38	0.018
	**Binary**	0.902	0.041	0.910	0.034	0.015	0.836	0.063	0.845	0.056	0.004
**Abundance**	**Bray**	0.968	0.069	0.982	0.042	0.058	0.926	0.136	0.942	0.114	0.085
	**JSD**	0.649	0.071	0.664	0.045	0.024	0.594	0.144	0.617	0.115	0.006
**Phylogeny**	**UniFrac**	0.810	0.062	0.813	0.062	0.180	0.768	0.105	0.775	0.097	0.220
	**wUniFrac**	0.612	0.089	0.617	0.090	0.262	0.425	0.156	0.449	0.157	0.031

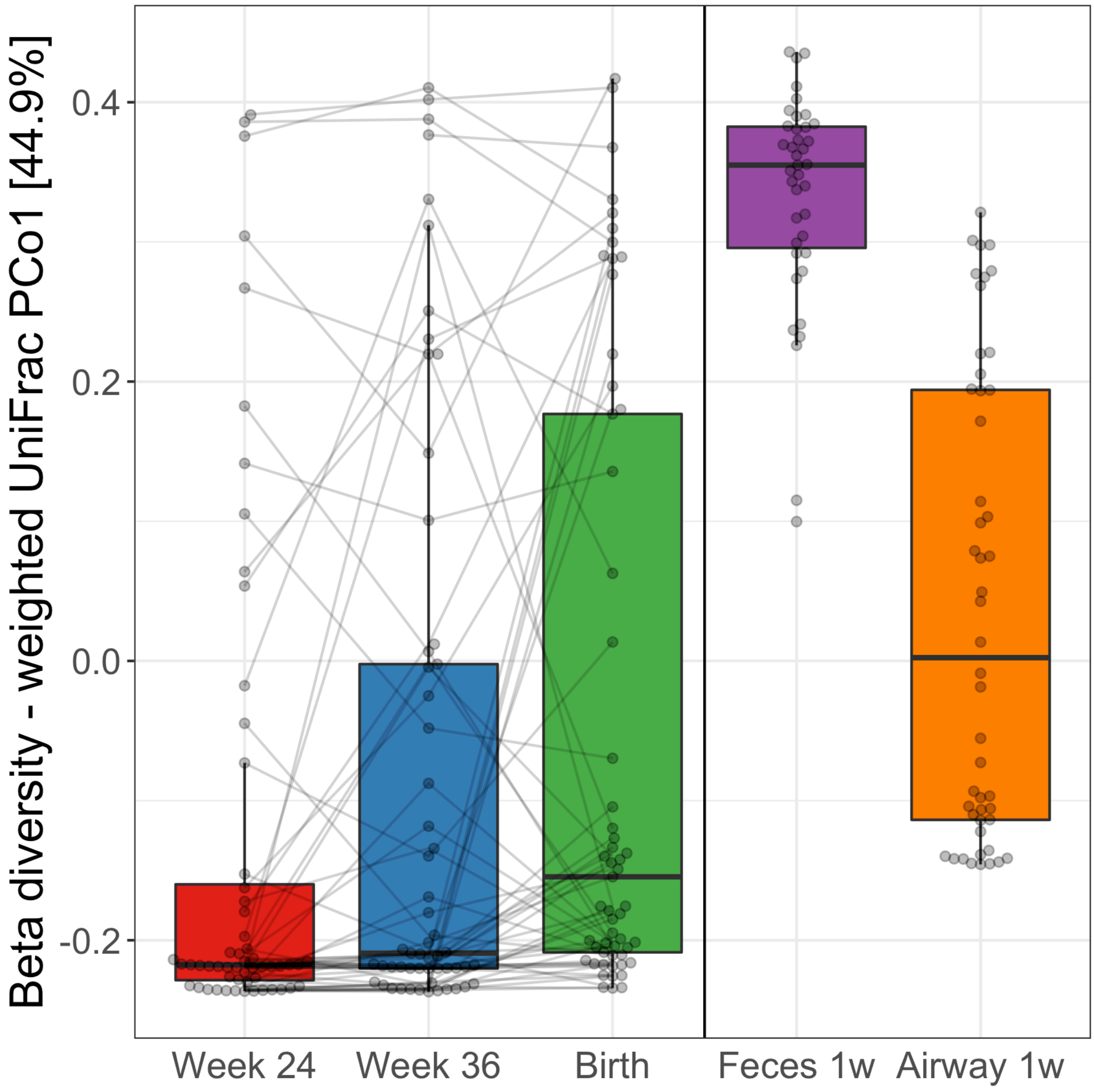

Figure S1 *– Principal Coordinate 1 of weighted UniFrac distances show a development towards a more fecal like composition towards the end of pregnancy and during birth, a similar trend to the unweighted UniFrac ordination used in Figure 2C.*

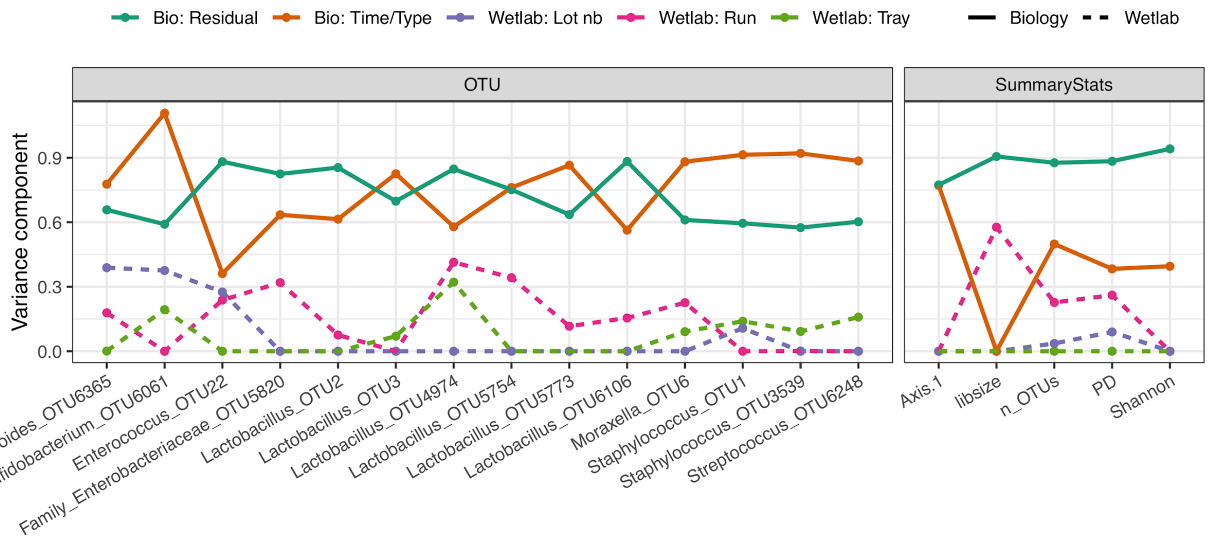

Figure S2 - *Variance distribution - in terms of standard deviations - of biological sources: Pregnancy week, child age and compartment (Bio: Time/Type) and residual (Bio: Residual), and wetlab sources: Lot number of Extraction Kit (Wetlab: Lot nb), Extraction tray (Wetlab: Tray) and Sequencing Run (Wetlab: Run), on the top 10 most abundant- and top 10 most present OTUs, and summary stats by first four axis of PCoA on weighted unifrac beta diversity, library size (libsize), number of OTUs present (n_OTUs), Faith's Phylogenitic Diversity (PD) and Shannon diversity (Shannon).*

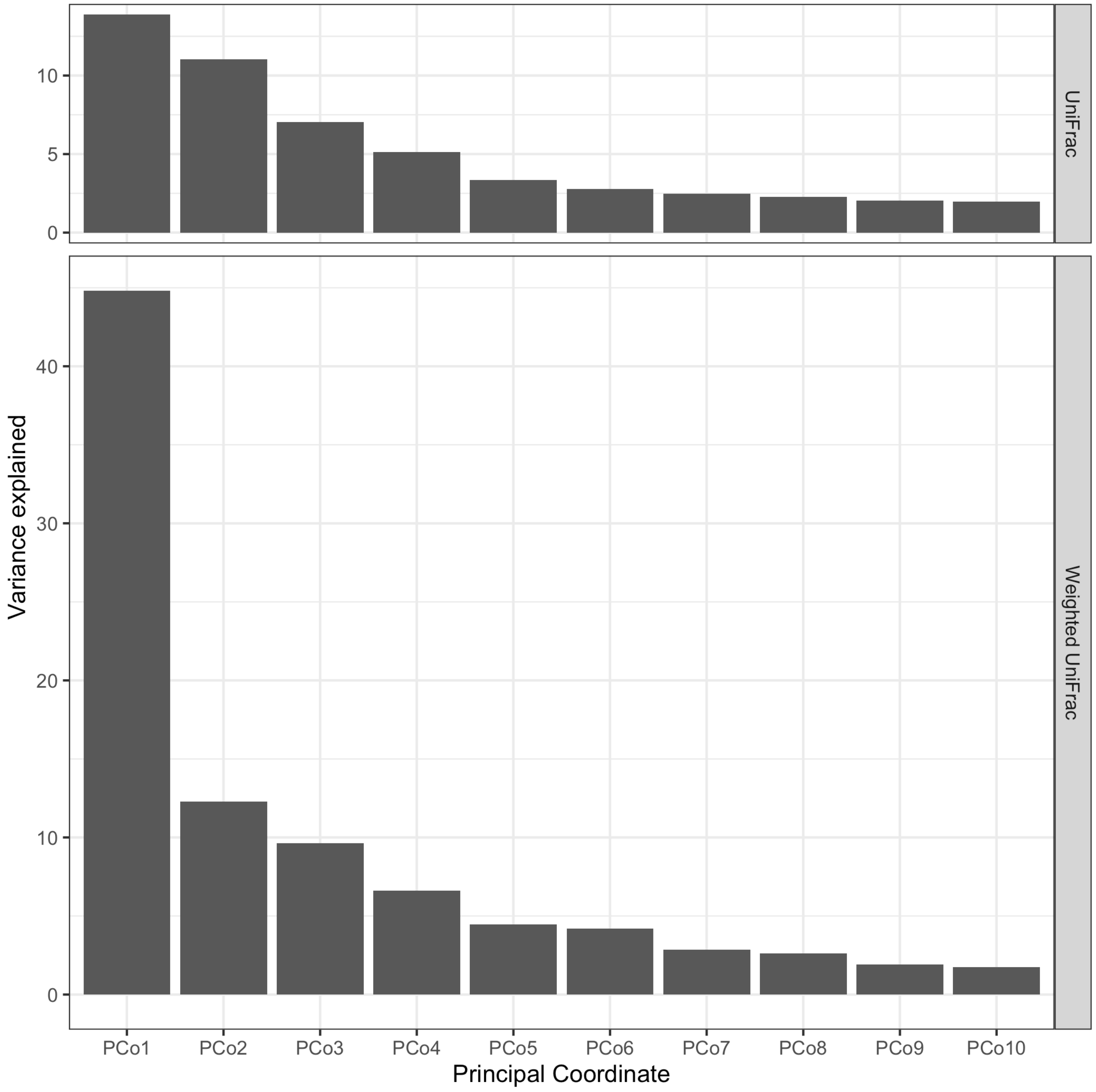

Figure S3 *– Scree plot of variance explained in UniFrac and weighted UniFrac ordinations on all samples, respectively.*

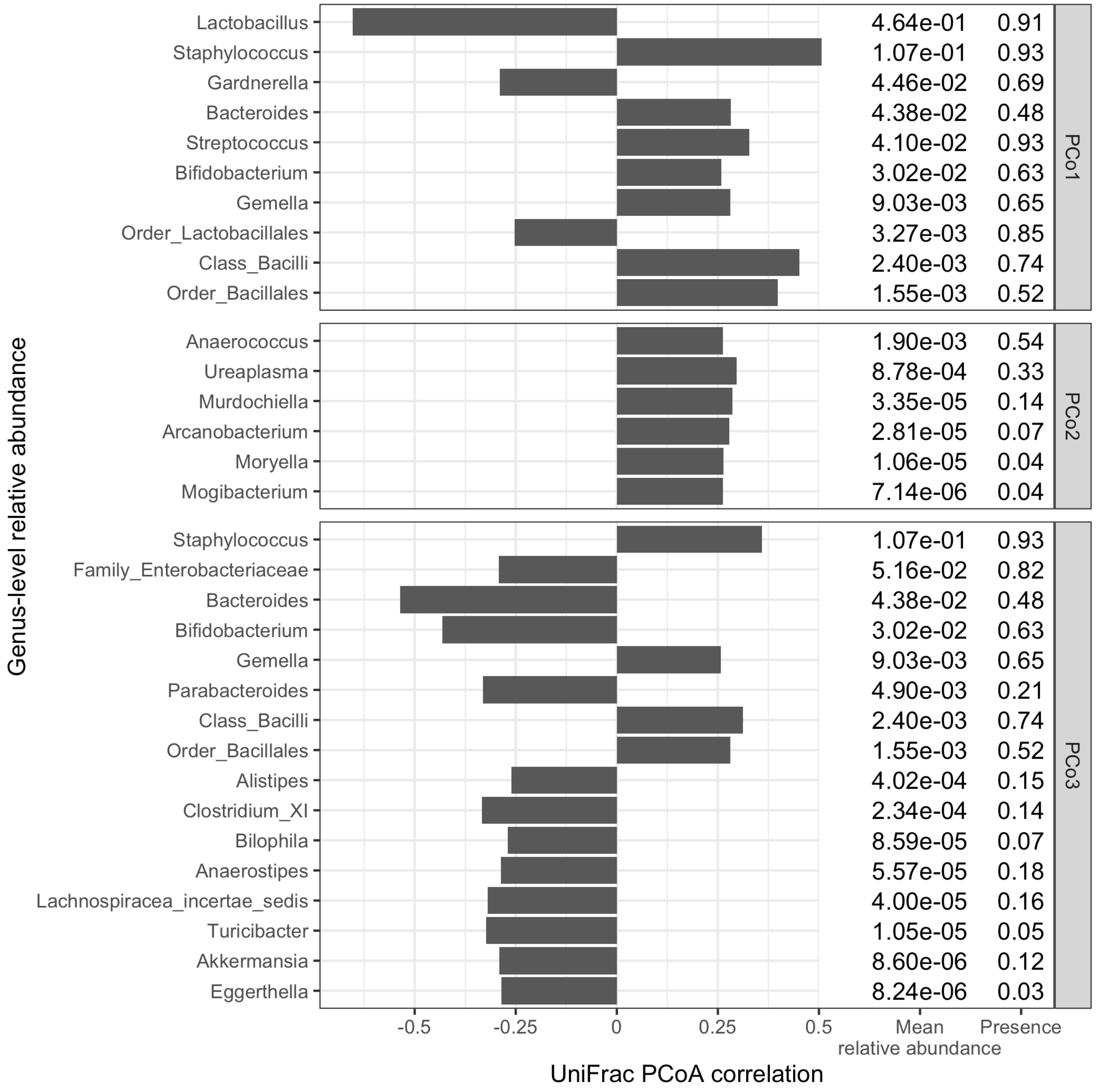

Figure S4 *– Post-hoc correlations between UniFrac Principal Coordinates 1-3 and genus-level relative abundances. Only values above 0.25 or below -0.25 are shown.*

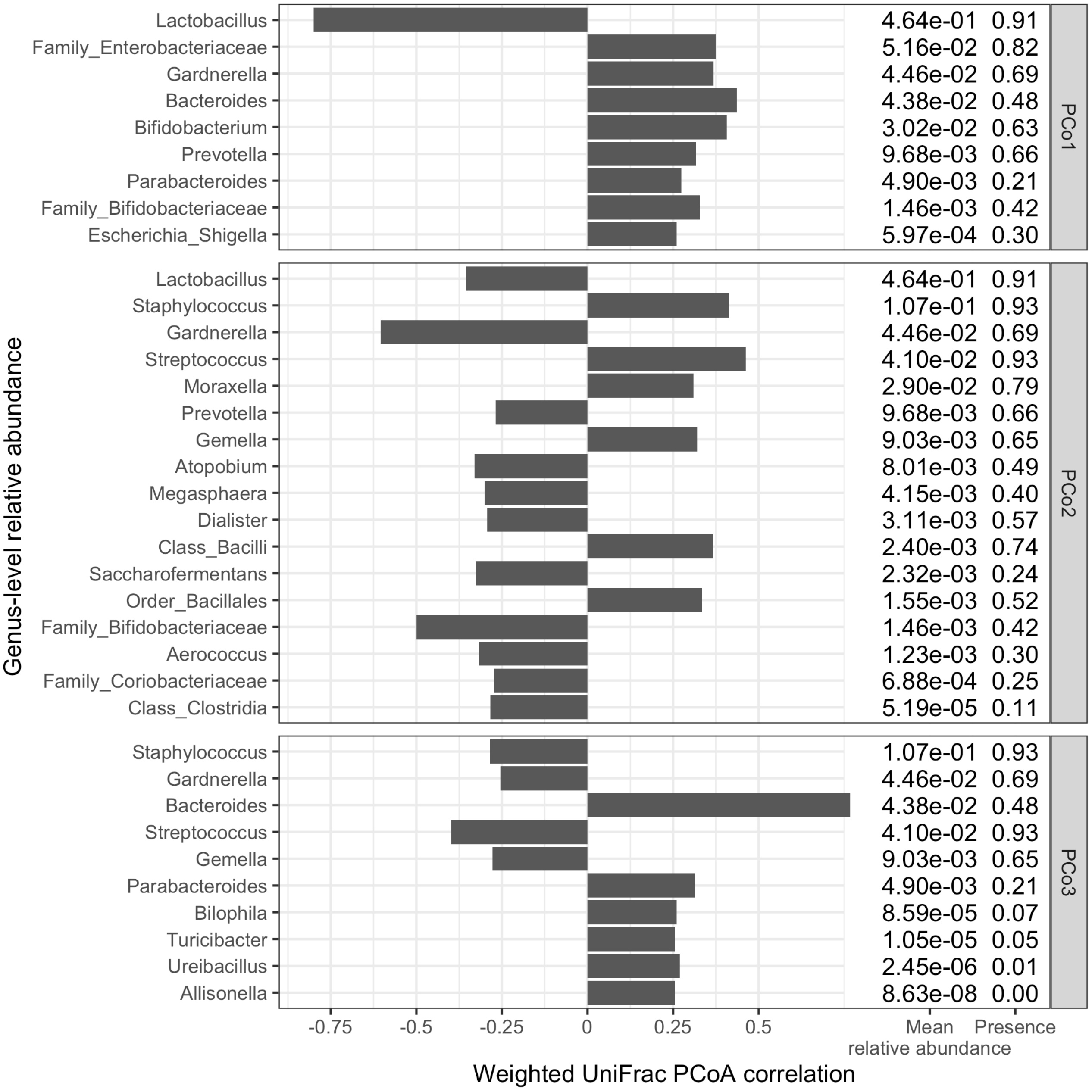

***Figure S5** – Post-hoc correlations between weighted UniFrac Principal Coordinates 1-3 and genus-level relative abundances. Only values above 0.25 or below -0.25 are shown.*

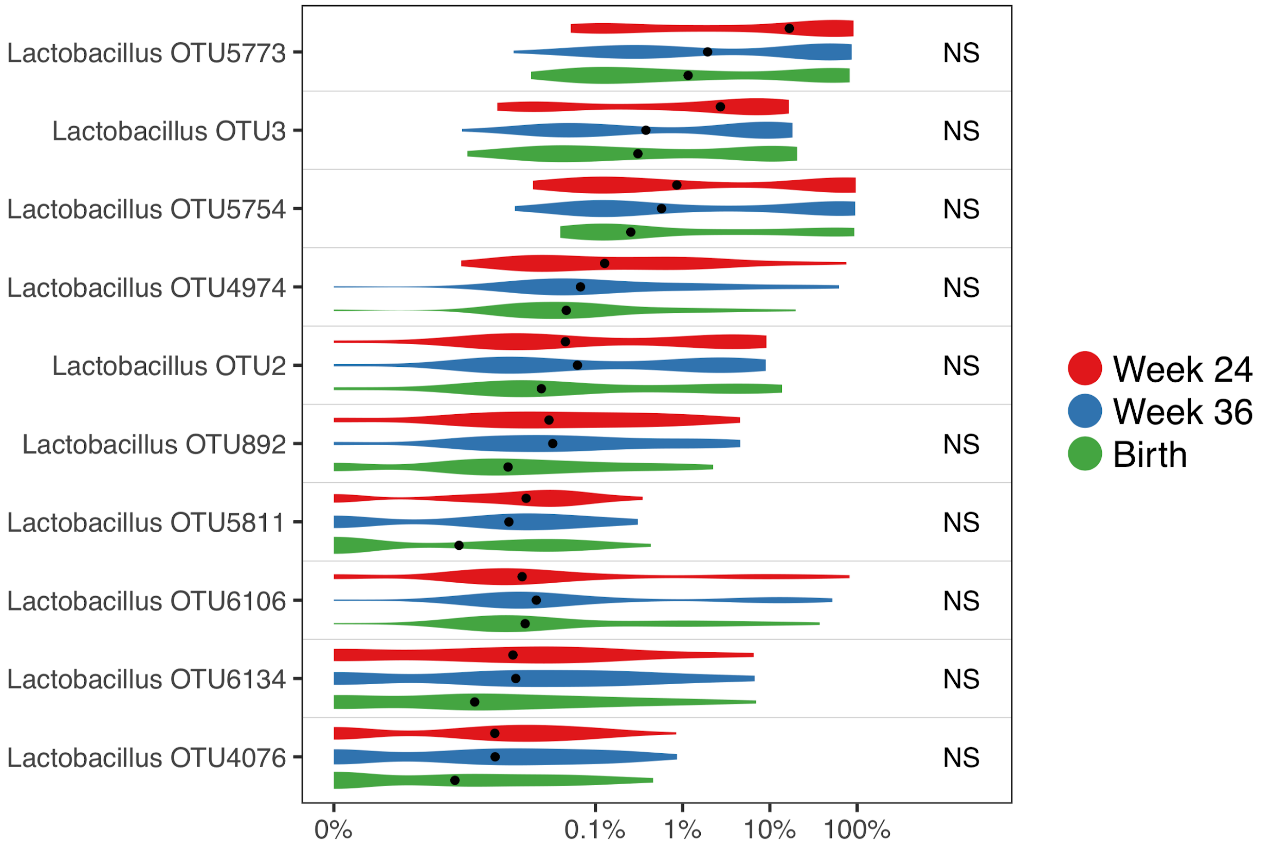

Figure S6 - *Relative abundance of the top ten dominating Lactobacillus OTUs tested for change during pregnancy.*

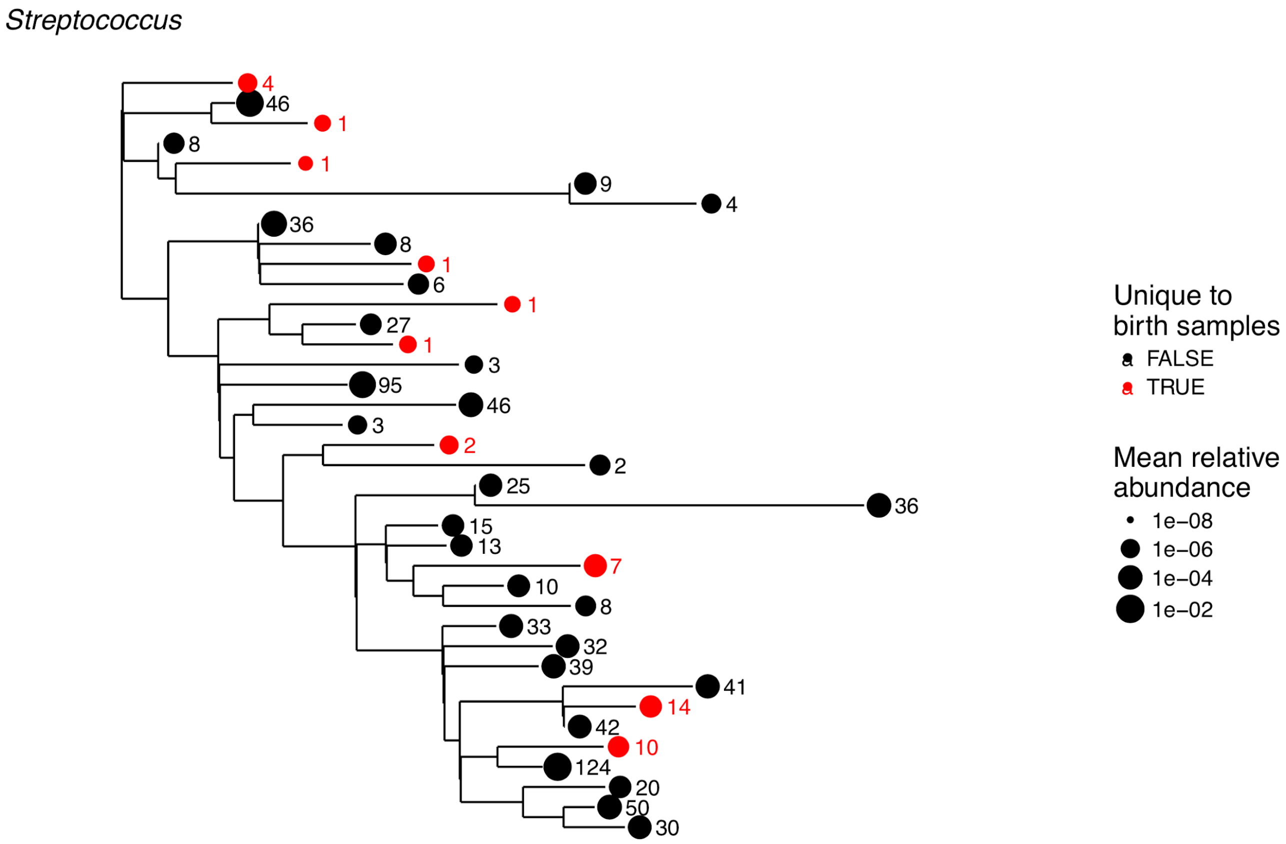

Figure S7 *- Phylogenetic placement of 37 Streptococcus OTUs highlighting the 10 observed only in the vaginal samples at birth after rupture of membranes. Labels on nodes indicate number of positive samples.*

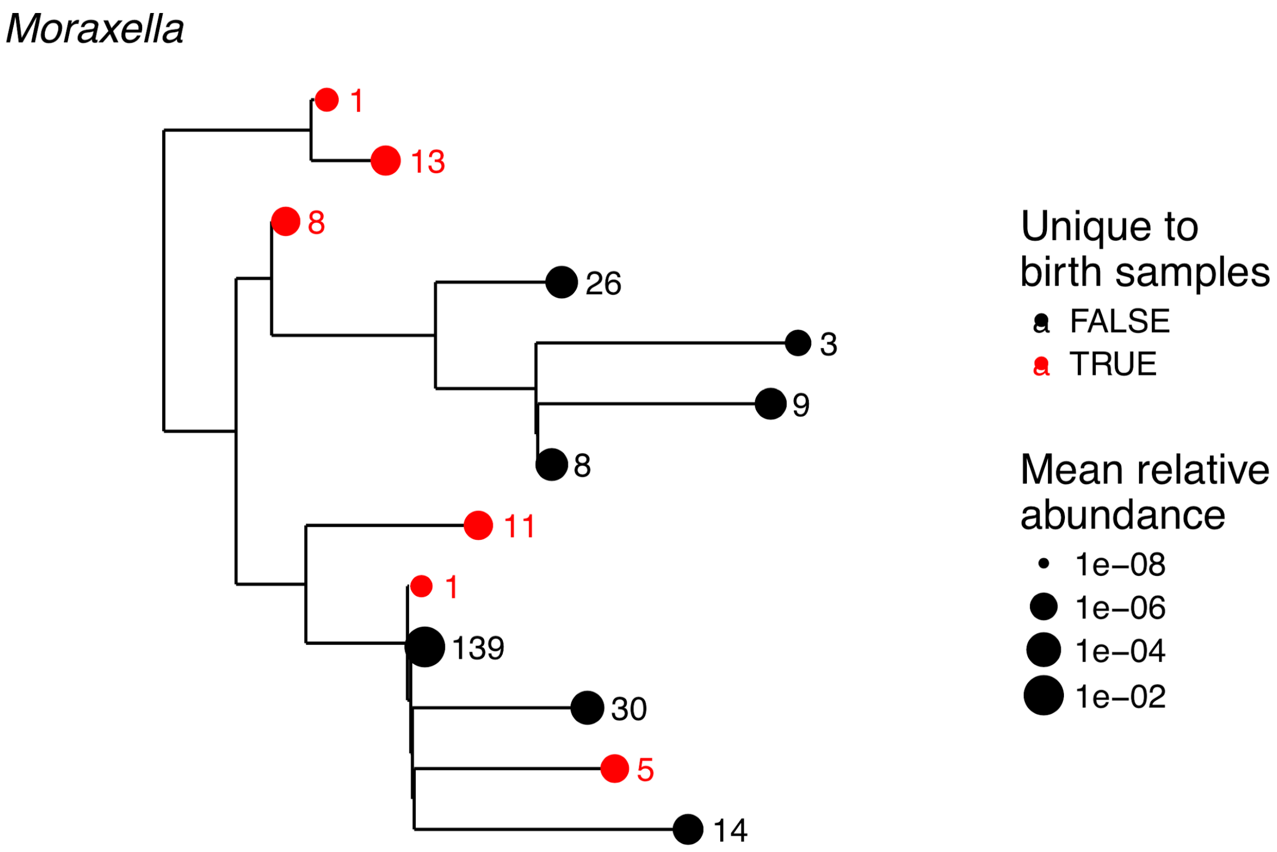

Figure S8 *- Phylogenetic placement of 13 Moraxella OTUs highlighting the 6 observed only in the vaginal samples at birth after rupture of membranes including a unique new branch. Labels on nodes indicate number of positive samples.*

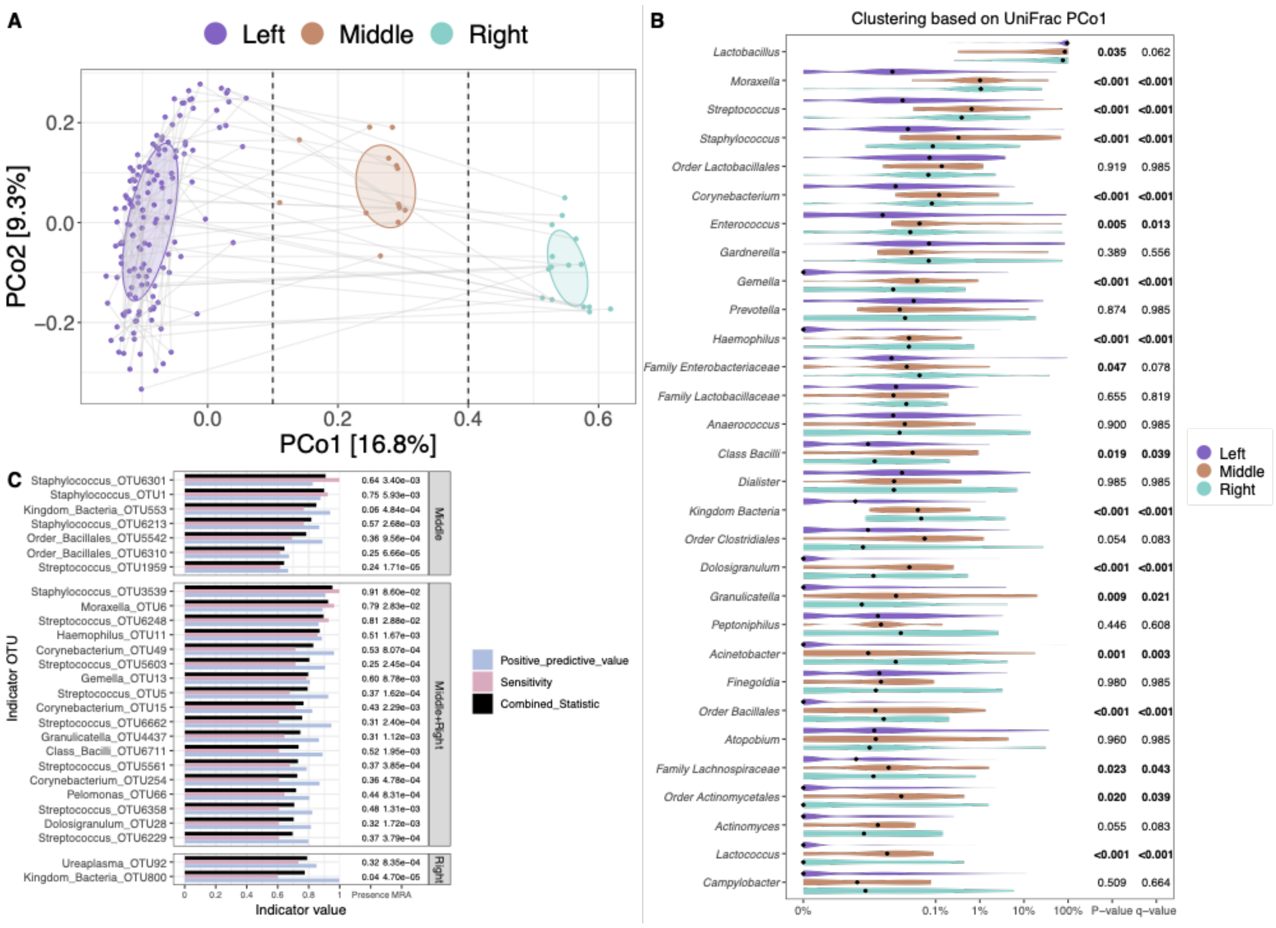

Figure S9 *- Subanalysis of Figure 4 examining the apparent grouping structure on PCo1.* ***A)*** *Only birth samples are in the middle and right clusters, delineated manually on PCo1, whereas the left cluster contains samples from all three timepoints. A decrease in the dominant Lactobacillus genus makes way for a relative increase in a large range of other taxa (****B****), with significant shifts as calculated by Kruskal-Wallis tests, with some taxa acting as sensitive and specific markers for the two other clusters or their combination compared to the left cluster (****C****), as calculated by the multipatt function from the R-package indicspecies.*

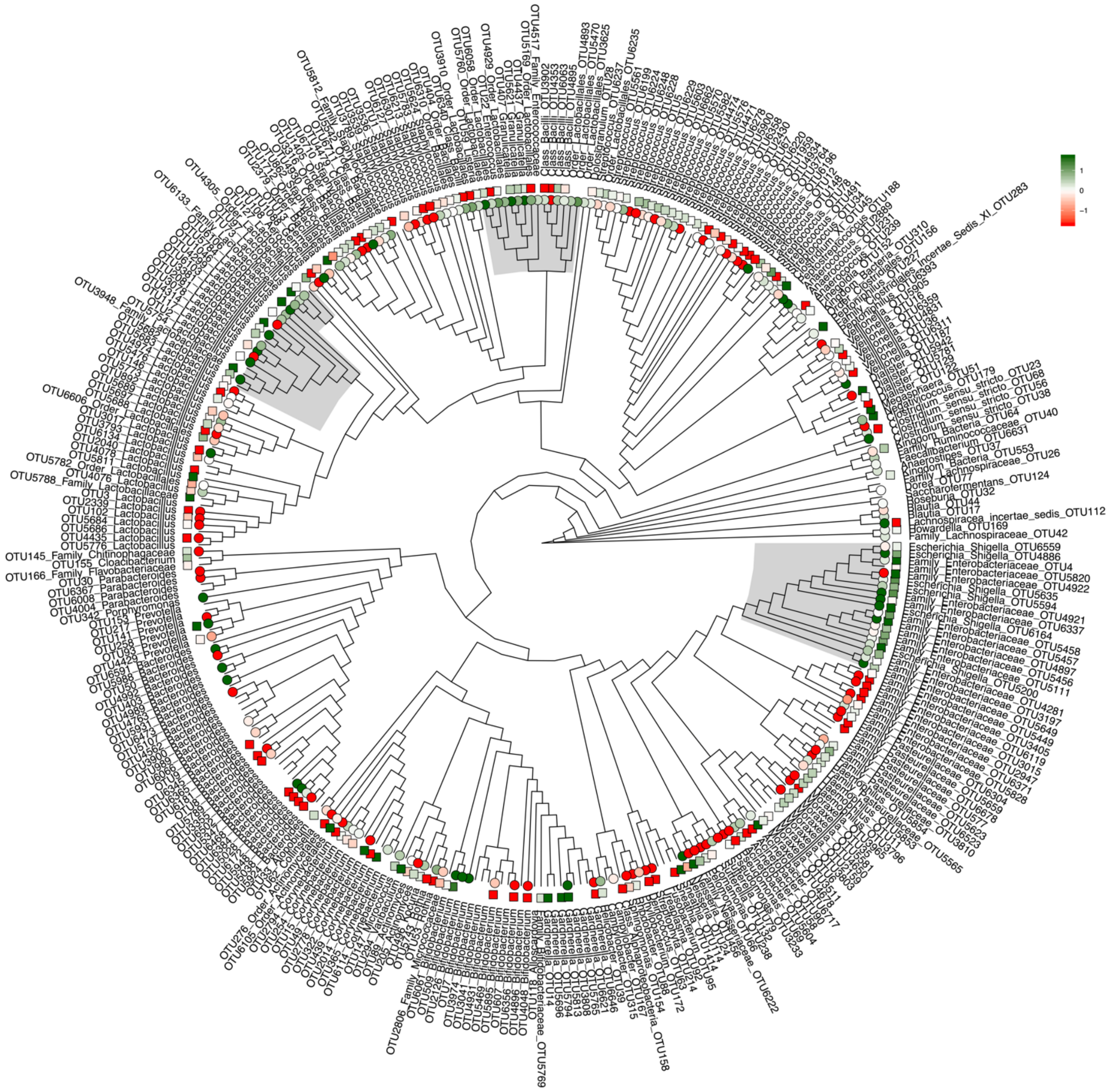

Figure S10 *- Phylogenetic tree of the 325 overall most common OTUs (present in more than 10% of the birth, fecal and airways sample). Odds ratios for vertical transfer from vagina to 1-week gut (circles) and airways (squares) for testable OTUs are inferred with color (green = positive odds, red = negative odds, log10 scale and truncated to 50 and 1/50 for positive and negative odds respectively). Highlighted are clades highly enriched with positive odds for transfer.*

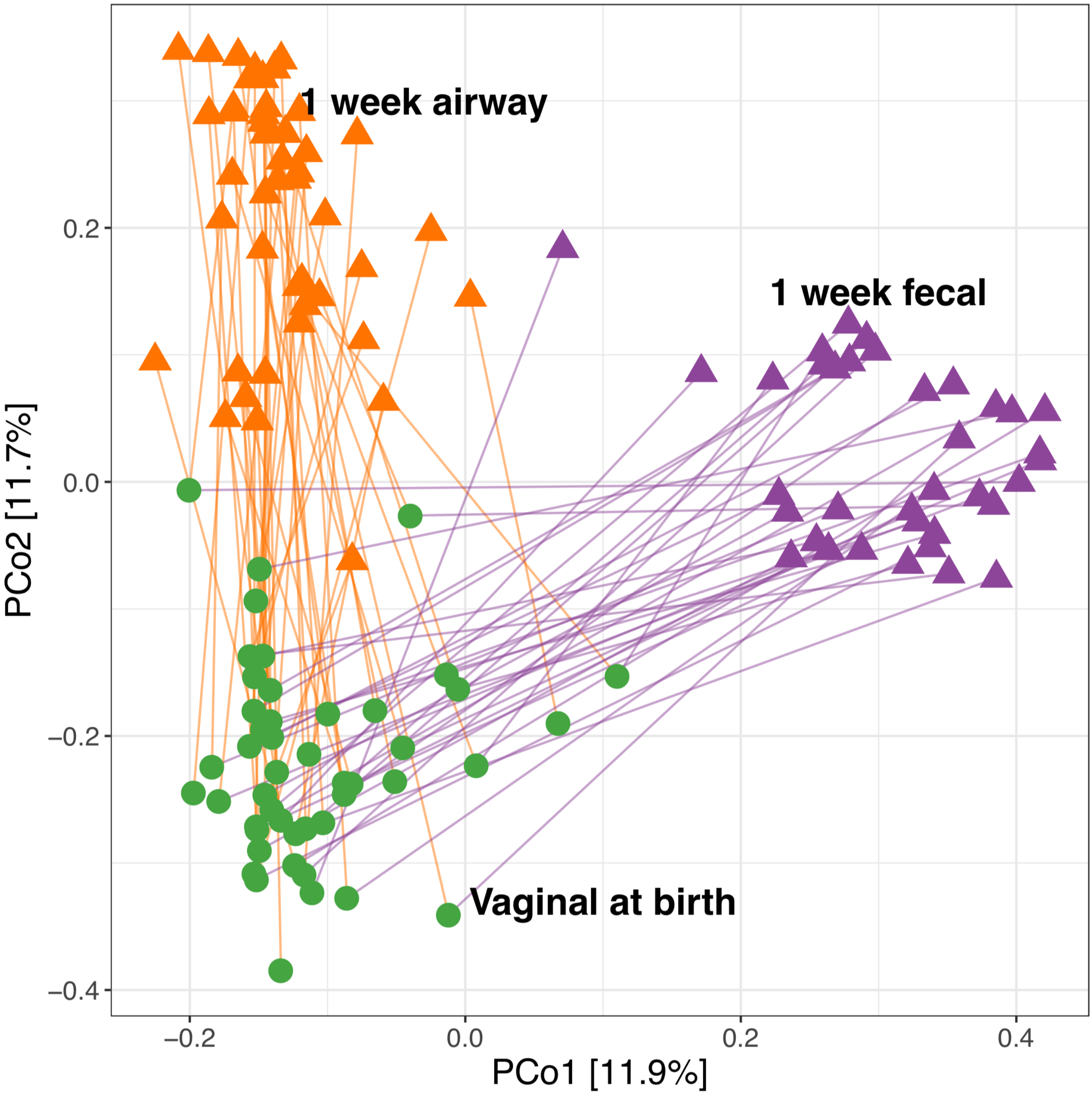

Figure S11 *- Ordination plot of binary comparison of microbiotas from vaginal samples at birth, 1-week fecal samples and 1-week airway samples. Connected within mother-child pairs.*
